# Genetic Differentiation between Resistance Phenotypes in the Phytophagous Flea Beetle, *Phyllotreta nemorum*

**DOI:** 10.1673/031.009.6901

**Published:** 2009-12-10

**Authors:** Peter W. de Jong, Casper J. Breuker, Helene de Vos, Kim M.C.A Vermeer, Keiko Oku, Patrick Verbaarschot, Jens Kvist Nielsen, Paul M. Brakefield

**Affiliations:** ^1^ Laboratory of Entomology, Wageningen University, PO Box 8031, 6700 EH Wageningen, The Netherlands; ^2^ Evolutionary Developmental Biology Research Group, School of Life Sciences, Oxford Brookes University, Gipsy Lane, Headington, Oxford, OX3 OBP, UK; ^3^ Institute of Biology Leiden, section Evolutionary Biology, University of Leiden, PO Box 9516, 2300 RA Leiden, The Netherlands; ^4^ Rice Bug Management Research Team, National Agricultural Research Center, Tsukuba, lbaraki 305-8666, Japan; ^5^ Department of Natural Sciences/Bioorganic Chemistry, University of Copenhagen, Thorvaldsensvej 40, DK-1871 Frederiksberg C, Denmark

**Keywords:** host race, allozymes, polymorphism, genetic structure, sympatric speciation, Mantel test, Barbarea vulgaris

## Abstract

The flea beetle *Phyllotreta nemorum* L. (Coleoptera: Chrysomelidae) is genetically polymorphic for resistance against the defences of one of its host plants, *Barbarea vulgaris* R.Br. (Brassicales: Brassicaceae). Whereas resistant flea beetles are able to use *B. vulgaris* as well as other cruciferous plants as food, non-resistant beetles cannot survive on *B. vulgaris*. This limitation to host plant use of non-resistant beetles could potentially lead to asymmetric gene flow and some degree of genetic isolation between the different resistance-genotypes. Therefore, we studied the extent of genetic differentiation at neutral allozyme loci between samples of flea beetles that were collected at different locations and first tested for resistance phenotype. Since earlier work has shown a weak, but significant, effect of geographical distance between the samples on their genetic differentiation, in the present study variation at the neutral allozyme loci in *P. nemorum* was partitioned between geographical distance and resistance-phenotype. Both sources independently contributed statistically significantly to population differentiation. Thus, there appears to be a limitation to genetic exchange between the resistant and non-resistant flea beetles when corrections are made for their geographic differentiation. This is consistent with the presence of some degree of host race formation in this flea beetle.

## Introduction

Amongst the most important, and perhaps most controversial, questions in biology is that of speciation (e.g. Barton 2001). Biological species evolve from lineages between which a barrier to genetic exchange has been established (Coyne 2007). The process of allopatric speciation is widely accepted, in which this barrier is between different geographical areas. In this case, geographical structure leads to a degree of genetic population structure, which, if large enough, may ultimately lead to genetically different lineages that no longer successfully interbreed (e.g. Turelli et al. 2001). There are, however, other ways that may lead to genetic structure within populations, and thus, potentially, speciation. A much more controversial mechanism is sympatric speciation (Via 2001; Berlocher and Feder 2002; Bolnick and Fitzpatrick 2007; Coyne 2007). This type of speciation does not require geographic separation between lineages. It may theoretically result from the establishment of an evolutionary novelty, such as a mutation influencing habitat use or mate choice (e.g. Jiggins 2008).

One example where convincing evidence has been obtained for genetic differentiation between sympatric lineages is the host plant use of phytophagous insects (Berlocher and Feder 2002; Drès and Mallet 2002; Dambroski et al. 2005; Egan et al. 2008; Xie et al. 2008). Here, in several cases, part of an insect population has acquired the ability to exploit a novel host plant species. Once such an evolutionary novelty has arisen, barriers to genetic exchange between insects using the ‘old’ host plant(s) and those able to use the new one as food may be generated through, for example, phenological constraints (Feder and Filchak 1999; Filchak et al. 2000; Dambroski and Feder 2007), pleiotropic effects (Mackenzie 1996), host preference (e.g. Forbes and Feder 2006) or assortative mating (Malausa et al. 2005). Thus, host plant races may evolve in the phytophagous insect that may, given enough time, lead to sympatric speciation.

Whereas, in the view of sympatric speciation geographical disjunction is not required, this does not imply the total absence of a geographical component in the precise shaping of the evolutionary pathway. It has been shown in many organisms that populations are genetically structured, e.g. through geographical discontinuities (see e.g. Mopper and Strauss 1998). Although genetic differentiation, for example in host plant races of phytophagous insects, may locally arise sympatrically, further, more global, spread of adaptations involved in host race formation may still be influenced by processes of migration, selection and drift. The eventual geographic distribution of adaptations involved in host plant use may thus be influenced by local differences in selection, as well as insect mobility. Thus, to understand the variation in host plant use by a phytophagous insect, it is crucial to detect the different factors that may limit the genetic exchange between subpopulations. This is the subject of the present paper, in which we report factors associated with population structure in the flea beetle *Phyllotreta nemorum* L. (Coleoptera: Chrysomelidae) and propose an explanation for the present-day distribution of its adaptation to an atypical host plant, *Barbarea vulgaris* ssp. *arcuata* R. Br. (Brassicales: Brassicaceae).

*P. nemorum* is an oligophagous flea beetle, feeding on a relatively narrow range of cruciferous host plants, most notably *Sinapis arvensis* L. (Brassicales: Brassicaceae) (Nielsen 1997 a,b). In East Denmark individuals have been found that used the atypical (though locally abundant) host plant *B. vulgaris*. This plant species has two types, called P- and G-type (with pubescent and glabrous leaves, respectively) of which the latter is chemically defended against at least some phytophagous insects (Nielsen 1997a). Thus, the majority of *P. nemorum* cannot feed on this plant. However, some populations of *P. nemorum* were found feeding on the G-type of *B. vulgaris* in East Denmark (Nielsen 1997a,b; de Jong and Nielsen 1999). These turned out to be genetically resistant to the plant defences. Whereas the resistant beetles can use the G-type of *B. vulgaris* as well as other host plant species that were studied, beetles that did not contain the resistance allele(s) could use the same range of host plants, except the G-type of *B. vulgaris*. This could represent a unilateral barrier to gene flow, namely from beetles using other plants than *B. vulgaris* (G-type) to those using this toxic plant.

Earlier work has shown that, whereas flea beetles sampled on the G-type of *B. vulgaris* were all resistant, the proportion of resistant beetles found on other host plants was always much lower, with a maximum of approximately 70% (de Jong and Nielsen 1999; Nielsen and de Jong 2005). Although an allozyme study indicated that there is some limitation to gene flow in *P. nemorum* due to the geographical distance between the samples, it was concluded that this barrier was unlikely to explain the geographical distribution of resistance in this beetle (de Jong et al. 2001). However, the existence of host races, partly isolated by unidirectional migration, and potentially also by other factors limiting gene flow of beetles from *B. vulgaris* to other host plants (e.g. phenological differences across host plants, preference, assortative mating, or selection against resistant migrants towards other host plants) could explain the geographical distribution of resistance. This could also represent an early step in sympatric speciation. In the present study we investigated whether there was any evidence for genetic diversification between resistance-phenotypes of *P. nemorum*, when corrections were made for effects of geographical distance between the samples.

## Materials and Methods

### Sampling and rearing

**Figure 1.**
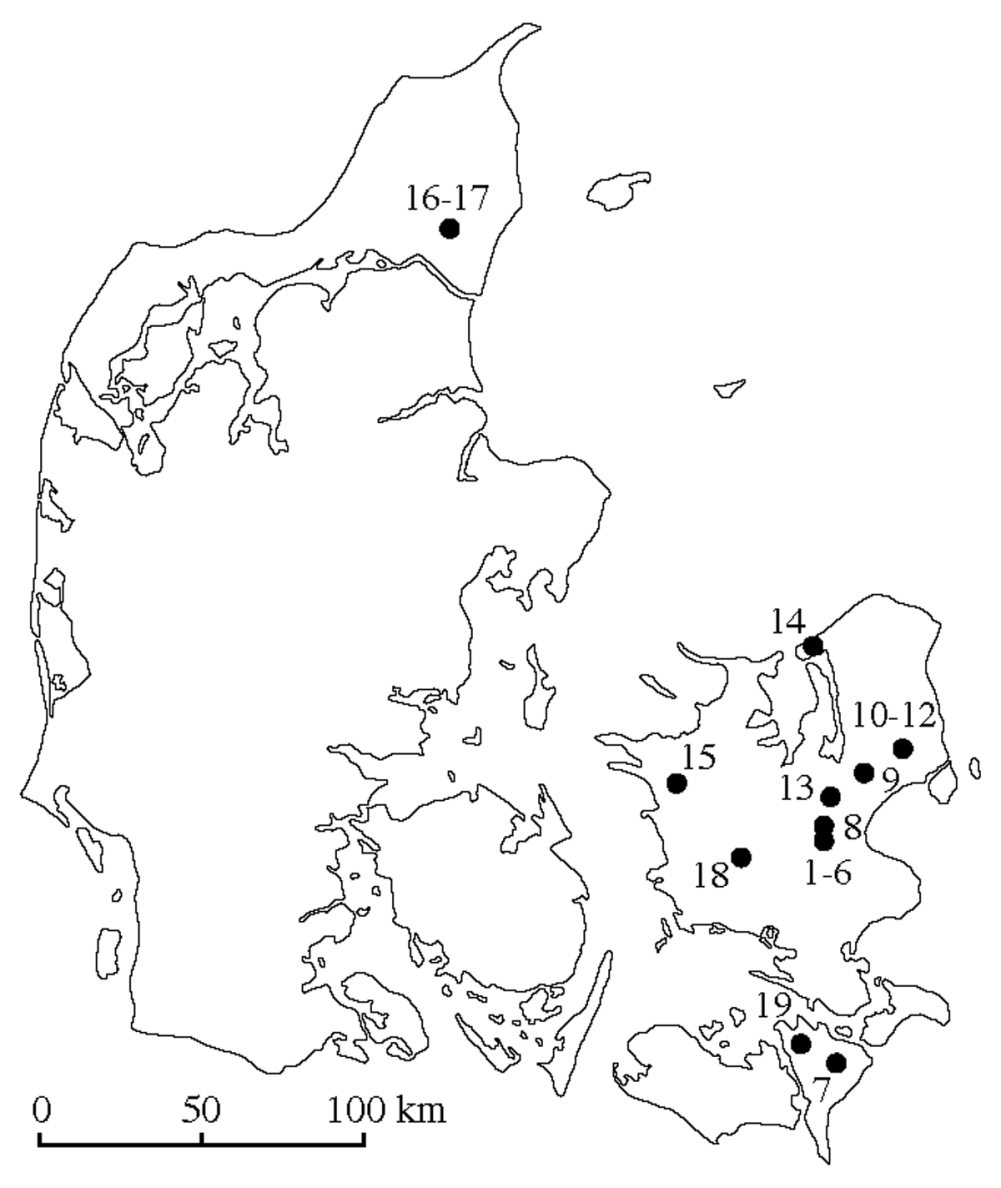
Locations in Denmark where samples were collected. The numbers refer to the sampling locations. In some cases, a number of samples were collected close together at one location; these are then indicated by one dot on the map. 1–6: Kværkeby; 7: Maglebrænde; 8: Vigersted; 9: Taastrup; 10–12: Ejby; 13: Viby; 14: Lynæs; 15: Svebølle; 16–17: Try Enge; 18: Suserup; 19: No. Vedby. Locations 13, 16–17, and 19 were not used for the analysis.

During June-July 1999 and 2000, flea beetle larvae were collected on different plants at 14 Danish localities by picking leaves containing leaf-mining larvae. At some locations samples were taken at multiple local plant patches, which were treated as distinct samples; they included 6 patches from Kværkeby, 2 from Ejby, 1 from Taastrup, Vigersted, Svebølle, Suserup, Lynæs, and Maglebrænde (Figure 1 and Table 1) (see also Nielsen and de Jong 2005). Leaves were picked from as many plants per sample as possible (as opposed to many from one plant) to avoid bias due to family structure. The leaves were kept in plastic bags at 20° C and a 18:6 L:D photoperiod, keeping the samples from different plants and/or localities separate. The bags were inspected at least daily (usually twice per day) for final instar larvae, which exit the mines. These were transferred to a plastic jar (500ml) closed with a lid having a cotton-plugged hole (diameter 1.5 cm). The jar contained a 5 cm layer of a mixture of moist peat and medium grain vermiculite in which the larvae pupated. Clean leaves, that were kept refrigerated before use, were added to allow additional larval feeding before pupation. When all larvae had burrowed into the vermiculite, the leaves were removed, and the jars were monitored daily for emerging adults. Subsequently, non-choice bioassays were performed with neonate beetles before they had had any access to food to determine their resistance-phenotype as described in de Jong and Nielsen (1999). Non-resistant *P. nemorum* do not accept *Barbarea* for feeding, whereas resistant individuals do (de Jong and Nielsen 1999).

### Bioassays

*B. vulgaris* seeds were collected in Herlev in 1994 (accession 3; Nielsen 1997a), and sown in a peat-vermiculite (grade 3) mixture (5:1). Plants were grown at 20 ± 2° C and a 18:6 L:D photoperiod (400W HPI/T lamps). Leaves from plants in the vegetative stage were used for bioassays.

Bioassays were performed in plastic vials (158 ml.) with a moist charcoal-gypsum bottom layer and closed with a plastic lid with a cotton-plugged hole (Nielsen 1978). Two *B. vulgaris* leaf discs (diameter 1.4 cm) were attached to the bottom of each vial with pins. One flea beetle was released per vial. After three days, the leaf discs were categorised into two classes: largely intact, and damaged by feeding. In this way beetles were categorised into nonresistant, and resistant individuals, respectively. This resulted in 22 samples from the 14 localities, 15 containing resistant, and seven containing non-resistant flea beetles. Some localities only had *B. vulgaris*, and hence only the resistant flea beetle phenotype. Subsequently, all beetles were fed with radish leaves for at least one day, and transported to the University of Leiden. No food was provided during transportation (c. 2 days). Upon arrival, the beetles were immediately frozen alive at -80° C, where they were kept until electrophoresis.

### Electrophoresis

Electrophoresis was performed following de Jong et al. (2001). Our previous study revealed enzyme-loci that could be reliably scored, were polymorphic in *P. nemorum*, and did not show any evidence of linkage or deviation from Hardy-Weinberg equilibrium (de Jong et al. 2001). The loci used in the present study, were (E.C. numbers in parentheses): aspartate aminotransferase (AAT;2.6.1.1), isocitrate dehydrogenase (IDH; 1.1.1.42), malate dehydrogenase (MDH1 and MDH2;1.1.1.37), and phosphoglucomutase (PGM;5.4.2.2). Samples were applied cathodally on the gels. MDH and IDH were run in Trismaleate buffer, AAT and PGM in Tris-glycine.

### Analysis

The data were analysed using GDA-software (Lewis and Zaykin 1999, version D12; based on Weir 1996). Tests were performed for Hardy-Weinberg equilibrium and linkage disequilibrium between loci. Exact significance was calculated using permutation tests (Fisher's exact test, 3200 runs).

Samples were compared pairwise, to produce a matrix with values of theta (**φ**), which is an index for the differentiation between the samples. Two other matrices were produced: one involving the pairwise geographical distances, and one indicating whether the samples were of the same (coded with ‘0’), versus different (‘1’) resistance-phenotype (following Goslee and Urban 2007). Correlations between matrices were calculated (partial Manteltests; ZT-software; Bonnet and Van de Peer 2002; see also Legendre 2000; Raufaste and Rousset 2001), testing for the influence of resistance-phenotype on **φ**, while controlling for the effect of geographical distances between the samples. Goslee and Urban (2007) note that a significant Mantel statistic is much lower than what one could expect from a conventional correlation analysis. Hence, a relatively low value of r should not be interpreted as a correlation of low biological significance, particularly when one of the matrices (such as that of the resistance-phenotype) is filled with categorical data (‘0’ and ‘1’, in this case).

## Results

### Distribution of different resistance-phenotypes

In all samples collected on *B. v8.4x10-3ulgaris*, the proportion of resistant *P. nemorum* ranged from 98–100%. Samples collected on other plants, even those less than 1 km. from *B. vulgaris*, usually contained many fewer resistant beetles (15.0–71.4%; see Nielsen and de Jong 2005). One exception involved two samples that were collected in Ejby: one on *B. vulgaris*, and the other on *S. arvensis* growing next to it. Here, the percentage of resistant beetles on *B. vulgaris* was 99.0%, and on *S. arvensis* 96.0%.

### General description of samples

The average size of the 22 samples was 31.1. The average proportion of polymorphic loci (99% criterion) across the samples was 0.82. The average number of alleles per locus was 2.98 (per polymorphic locus 3.44). The mean proportion of heterozygotes across all samples was 0.23 (expected: 0.23).

**Table 1.**
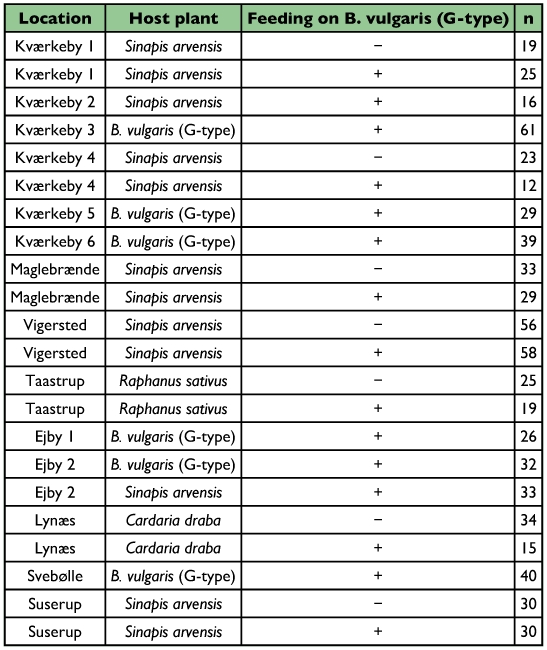
The 22 samples collected at the 14 different localities in East Denmark, including the host plant on which collections were made, resistance phenotype of each sample (+: resistant to defences of B. vulgaris), and the number of beetles that were used for the analysis.

### Hardy Weinberg equilibrium and linkage disequilibrium

With the exception of one locus (IDH) in one small (n=16) sample, no deviations from Hardy Weinberg equilibrium were detected for any of the loci in any of the samples (Fisher's exact tests; sequential Bonferroni for five loci per sample). In that single sample, significant linkage disequilibrium was found between IDH and the other four loci (Fisher's exact tests, 3200 runs with shuffling; P<8.4×10-3 ; Bonferroni correction for 10 tests per sample). If the significance level was adjusted for all performed tests across all samples, no significant deviation from Hardy Weinberg equilibrium, and no linkage disequilibrium was found. Therefore, it was assumed in further analysis that all loci were in Hardy Weinberg equilibrium in all samples, and there was no linkage disequilibrium between loci.

### Correlations between φ, geographical distance, and resistance-phenotype

**Figure 2.**
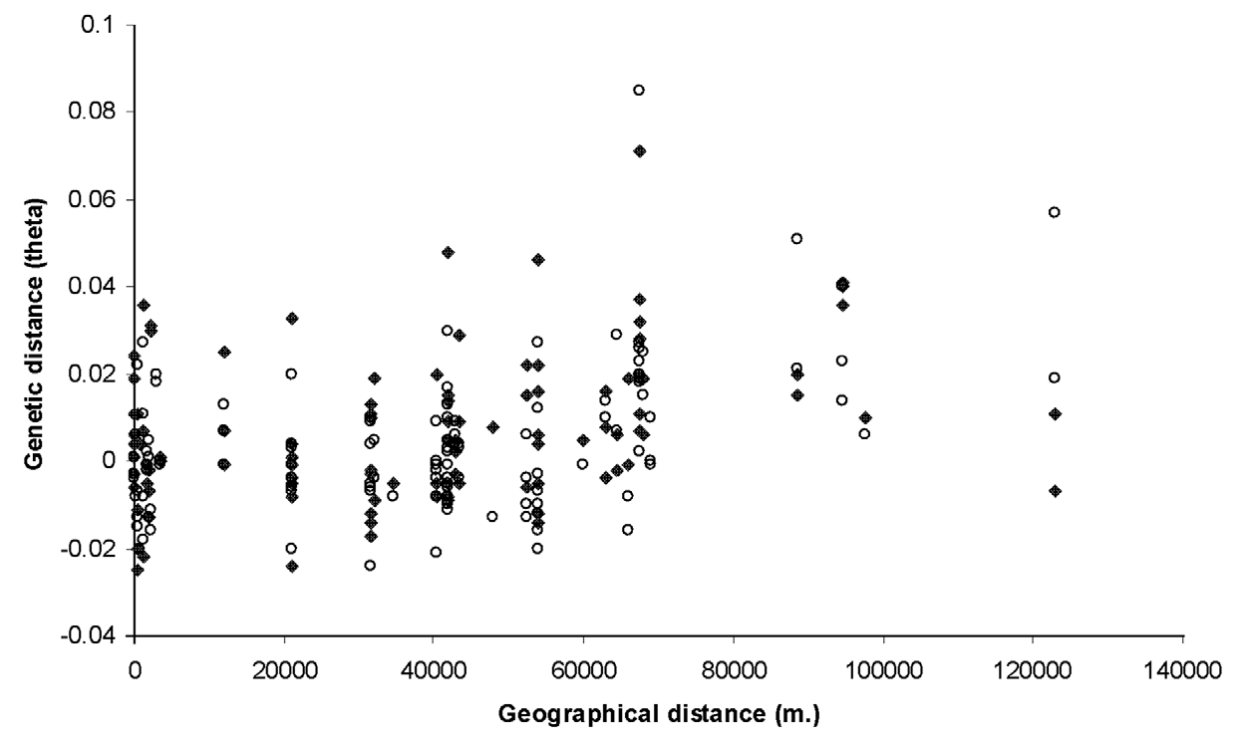
Pairwise genetic distance (theta) against pairwise geographical distance. Closed symbols: comparison between different resistance phenotypes; open symbols: comparison between identical resistance phenotypes.

The relationship between **φ** and geographical distance for pairwise comparisons of samples of the same phenotype, and between different resistance-phenotypes, revealed the typical pattern emerging when there is isolation by distance between populations: a positive correlation between geographical distance and **φ**, with an increasing variation of **φ** with larger distances (Figure 2). The positive relationship between **φ** and geographical distance was statistically highly significant (Mantel test, 100000 randomisations, r = 0.375, P = 0.00001), also when corrected for beetle phenotype (Partial Mantel test, 100000 randomisations, r = 0.379, P = 0.00001; see Materials and Methods for comments regarding the value of r of the Mantel test). After correction for this influence of geographical distance, the resistance-phenotype of the beetles was also significantly positively correlated with **φ** (Partial Mantel test with correction for effect of geographical distance; 100000 randomisations, r = 0.131, p=0.024), with higher between than within phenotype **φ**values. Thus, geographical distance and beetle phenotype independently contribute significantly to variation in the amount of genetic differentiation between the samples.

## Discussion

These results reveal two independent sources of genetic differentiation in the flea beetle *P. nemorum*: geographical distance (which was earlier found by de Jong et al. 2001) as well as resistance phenotype (which had not been studied before). Despite the limited allelic variability at the allozyme loci used in this study, and therefore the low resolution of population differentiation, pairwise theta values between samples containing different resistance phenotypes were statistically significantly larger than those comparing the same phenotype, after correction for the effect of geographical distance. This result implies that the genetic exchange between different phenotypes is limited, independently of isolation by distance.

An earlier study (de Jong and Nielsen 2001) concluded that no genetic differentiation was detectable between beetles collected on different host plants with overlapping local distributions. Those results involved comparisons between samples consisting of mixtures of resistance phenotypes (note that on other host plants than *B. vulgaris*, both resistant and non-resistant flea beetles occur). The present analysis, therefore, is the first rigorous test of the existence of genetic differentiation between beetles that can, and cannot, use *B. vulgaris* (G-type) as food. This may indicate the beginning of host race formation.

Various factors may be responsible for the genetic differentiation between different resistance-phenotypes of the flea beetles. First, non-resistant flea beetles cannot use the G-type of *B. vulgaris* as food. They are therefore more likely to mate with flea beetles living on other host plants than *B. vulgaris* that would have a mixture of non-resistant and resistant beetles. In this case, gene flow from nonresistant to resistant flea beetles would be partially impeded. Other factors that may further enhance the isolation between the resistance phenotypes include host preference, different host phenologies leading to asynchrony in host plant use by the different resistance phenotypes, or selection limiting gene flow from resistant to non-resistant flea beetles.

No evidence has been published so far supporting host preference in the flea beetles. If resistant flea beetles preferred to feed on the G-type of *B. vulgaris*, it would limit the gene flow from resistant to non-resistant flea beetles, leading to a larger degree of isolation between the phenotypes than that only caused by the inability of non-resistant individuals to feed on *B. vulgaris*, G-type.

There is some support for phenological effects that may affect genetic exchange between the phenotypes. *B. vulgaris* overwinters as a rosette, and is available immediately when the flea beetles emerge from overwintering in early spring. The other major host plant, *S. arvensis*, is an annual, and this plant becomes available later in the season after germination of the seeds (Nielsen 1997a). Thus, in early spring *B. vulgaris* (G-type) may be colonized by resistant flea beetles that would then mate assortatively, leading to a degree of temporal isolation between the phenotypes. Such phenological factors influencing host race formation have also been documented for the applemaggot fly (e.g. Feder and Filchak 1999).

Evidence has also been obtained for the third possible factor enhancing isolation between flea beetle resistance phenotypes, namely selection against resistant flea beetles. De Jong and Nielsen (2000) found a negative pleiotropic effect of resistance: homozygosity for resistance reduced the chance of survival in a crossing experiment with strains that had been reared in the laboratory for five generations. In the field, therefore, resistant migrants competing with non-resistant conspecifics on other host plants than *B. vulgaris* may be selected against (de Jong and Nielsen 2002). This would mean that genetic exchange between the resistance phenotypes is reduced by selection in two different directions: 1) non-resistant beetles are selected against when they attempt to feed on *B. vulgaris* (G-type), and 2) resistant beetles are selected against when they compete with non-resistant ones on other plants than *B. vulgaris*. To what extent the restriction in gene-flow due to selection at the resistance-locus is also reflected in neutral allozyme variation, depends on the timing of effects of selection relative to reproduction; gene-flow at neutral loci may be very high despite strong selection at an adaptive locus if neutral alleles can be established in the population before the effects of selection are manifested.

The possible causes of genetic differentiation between resistance-phenotypes in *P. nemorum* described above are not mutually exclusive. It is, however, likely, given the low estimates of genetic differentiation that were found in the present study and previous work (de Jong and Nielsen 2001), that a substantial amount of gene flow exists across resistance phenotypes and geographical distance. On the continuum of host plant races to (sympatric) species, therefore, the flea beetles are likely to be positioned at the very beginning of host plant associated differentiation.

The restriction of genetic exchange between the resistance phenotypes of *P. nemorum* as found in the present study helps understand the geographical distribution of resistant versus non-resistant beetles. Field populations of *P. nemorum* living on *B. vulgaris* G-type have all been found to be resistant to the plant defences. This is not surprising, since the individuals were always sampled in the larval stage, and these larvae are leaf miners with a restricted mobility (Alford 1999). They can only feed on the defended type of *B. vulgaris* if they are resistant to its defences. The interesting question with respect to the spatial distribution of resistance phenotypes of *P. nemorum* is: why are resistant larvae relatively rare on other plants than *B. vulgaris* (G-type)? In the absence of limitations to genetic exchange between beetles that are, and are not, able to use *B. vulgaris* (G-type), one would expect a rapid spread of the resistance trait in the flea beetles. The fact that a large proportion of flea beetles on other host plants than *B. vulgaris* are non-resistant to the defences of this plant can be understood by the barrier to gene flow that apparently exists across the resistance phenotypes. This barrier can be formed by any of the mechanisms described above, or combinations between them. The exact proportion of resistant to non-resistant flea beetles may vary locally, depending on the topographical and ecological circumstances influencing the amount of gene flow, e.g. proximity of patches of *B. vulgaris* G-type that are used by resistant flea beetles, relative abundance of different host plants, local differences in phenology of different host plants, and so forth. The global relationship between *P. nemorum* and its host plants is likely to be best described by a mosaic of local interactions between the beetles, their host plants and their enemies, leading to locally adapted subpopulations that are genetically isolated to some extent (cf. Thompson 2005). In a number of these localities, host race formation may be actually occurring. One of the first steps that should be followed now is to identify the variable mechanisms leading to the observed genetic differentiation between the resistance phenotypes of these flea beetles.
